# Supplementary material for: Deletion of wfs1 Impairs Oligodendrocyte Precursor Cells Dorsal Distribution and Myelination Through the wfs1-hmgcs1 Axis in Zebrafish
Source: Biology (Basel). 2026 Aug 16;15(16):1402. doi: 10.3390/biology15161402 (PMC13510151; doi:10.3390/biology15161402)
Supplement: Supplementary file 1 [file biology-15-01402-s001.zip › File S1-Transcriptome sequencing data and differentially expressed genes (DEGs) in wfs1-- zebrafish at 5 dpf/2.KEGG/wfs1KOvsWT/ALL/src/dre04340.html]

dre04340


- dre:100006858

- Down regulated genes

100006858\_1(-1.49071226984431)

- dre:323883

- Down regulated genes

323883(-4.8491500403135)

- dre:30269

- Down regulated genes

30269\_1(-1.97370033571951)

- dre:100006858

- Down regulated genes

100006858\_1(-1.49071226984431)

- dre:30269

- Down regulated genes

30269\_1(-1.97370033571951)

Close
